# Identification of Novel Influenza Polymerase PB2 Inhibitors Using a Cascade Docking Virtual Screening Approach

**DOI:** 10.3390/molecules25225291

**Published:** 2020-11-13

**Authors:** Lei Zhao, Jinjing Che, Qian Zhang, Yiming Li, Xiaojia Guo, Lixia Chen, Hua Li, Ruiyuan Cao, Xingzhou Li

**Affiliations:** 1 Beijing Institute of Pharmacology and Toxicology, 27 Taiping Road, Beijing 100850, China; leizhao-hit@hotmail.com (L.Z.); chejinjing80@126.com (J.C.); 15227117791@163.com (X.G.); 2 Department of Medicinal Chemistry, School of Pharmacy, Fudan University, Shanghai 201203, China; zhangqian511@shmu.edu.cn; 3 West China School of Medical, Sichuan University, Chengdu 610041, China; liyiming9947@163.com; 4 Key Laboratory of Structure-Based Drug Design and Discovery, Ministry of Education, Wuya College of Innovation, Shenyang Pharmaceutical University, Shenyang 110016, China; 5 Hubei Key Laboratory of Natural Medicinal Chemistry and Resource Evaluation, School of Pharmacy, Tongji Medical College, Huazhong University of Science and Technology, Wuhan 430030, China

**Keywords:** virtual screening, influenza virus, polymerase basic protein 2, inhibitor

## Abstract

To discover novel inhibitors that target the influenza polymerase basic protein 2 (PB2) cap-binding domain (CBD), commercial ChemBridge compound libraries containing 384,796 compounds were screened using a cascade docking of LibDock–LigandFit–GOLD, and 60 compounds were selected for testing with cytopathic effect (CPE) inhibition assays and surface plasmon resonance (SPR) assay. Ten compounds were identified to rescue cells from H1N1 virus-mediated death at non-cytotoxic concentrations with EC_50_ values ranging from 0.30 to 67.65 μM and could bind to the PB2 CBD of H1N1 with *Kd* values ranging from 0.21 to 6.77 μM. Among these, four compounds (11D4, 12C5, 21A5, and 21B1) showed inhibition of a broad spectrum of influenza virus strains, including oseltamivir-resistant ones, the PR/8-R292K mutant (H1N1, recombinant oseltamivir-resistant strain), the PR/8-I38T mutant (H1N1, recombinant baloxavir-resistant strain), and the influenza B/Lee/40 virus strain. These compounds have novel chemical scaffolds and relatively small molecular weights and are suitable for optimization as lead compounds. Based on sequence and structure comparisons of PB2 CBDs of various influenza virus subtypes, we propose that the Phe323/Gln325, Asn429/Ser431, and Arg355/Gly357 mutations, particularly the Arg355/Gly357 mutation, have a marked impact on the selectivities of PB2 CBD-targeted inhibitors of influenza A and influenza B.

## 1. Introduction

Influenza is an acute respiratory infection caused by influenza viruses, including human influenza viruses and animal influenza viruses. The influenza viruses capable of infecting humans are classified into types A, B, and C based on their core proteins [1]. Influenza A and B viruses are the primary influenza viruses that infect humans and cause seasonal epidemics of disease almost every year. There are 3 to 5 million cases of severe influenza disease worldwide each year, with an estimated 250,000 to 500,000 deaths [2]. As an extreme example, in the 1918–1919 Spanish flu pandemic, at least 20 to 40 million people worldwide died of the flu [3].

The variability of influenza virus antigens renders vaccines unable to provide permanent protection, and these vaccines cannot necessarily provide protection when new influenza subtypes appear. Hence, antiviral drugs are a type of crucial weapon in preventing and treating serious influenza infections, particularly in vulnerable individuals. To date, three types of approved antiviral drugs have been used: (i) inhibitors of the viral neuraminidase (NA), such as zanamivir and oseltamivir; (ii) compounds that block the matrix-2 (M2) ion channel, such as amantadine and rimantadine; and (iii) RNA-dependent RNA polymerase (RdRp) inhibitors, such as baloxavir and favipiravir. The M2 blockers are no longer recommended for treating seasonal influenza due to widespread drug resistance [3]. The neuraminidase inhibitors have only a moderate impact on the severity of flu symptoms and the duration of sickness, and these drugs must be administered within 24–48 h of infection to achieve noticeable results [4]. Furthermore, strains of oseltamivir- and zanamivir-resistant influenza viruses have been circulating for a long time [5,6], and a drug-resistant strain toward the newly approved baloxavir appeared soon [7,8]. The indications for another approved anti-influenza drug, favipiravir, is limited because of safety and other issues [9]. Consequently, there is an urgent need to find new antiviral drugs with novel mechanisms and no cross-resistance with existing drugs.

Influenza viruses, which belong to the Orthomyxoviridae group, are enveloped, segmented, single-stranded, and negative-sense RNA viruses. Each fragment of the viral genome forms a viral ribonucleoprotein (vRNP) consisting of multiple nuclear protein copies and a viral RdRp [10,11]. The viral RdRp consists of a heterotrimeric complex comprising polymerase acidic protein (PA), polymerase basic protein 1 (PB1), and polymerase basic protein 2 (PB2) subunits that are responsible for the replication and transcription of the viral genome in the nucleus of infected cells [12]. Transcription is a primer-dependent process; since the influenza virus cannot produce its own 5′-terminal 7-methylguanosine (m7G) cap primer, its 5′ cap primers are obtained via the so-called “cap-snatching process.” During this process, the viral RdRp uses its PB2 cap-binding domain (CBP) to capture the 5′ cap of nascent host capped RNAs, and its PA endonuclease domain cleaves the capped RNA approximately 8–14 nucleotides downstream of the cap structure. The PB1 subunit contains the conserved polymerase domain and utilizes this fragment as a primer for RNA elongation [13,14]. Because there is no mechanism similar to this cap-snatching process in human cells, the development of PB2 or PA inhibitors that disrupt the cap-snatching process is considered an ideal strategy for generating new anti-influenza drugs. To date, a PB2 inhibitor (VX-787) has completed phase II clinical trials and is undergoing further evaluation in phase III clinical trials [15,16]. However, the development of VX-787 is not progressing smoothly. One possible reason is that the pyrimidine-7-azaindole motif of VX-787 can be metabolized by human aldehyde oxidase (AO), resulting in a poor pharmacokinetic profile [17]. Thus, searching for a PB2 inhibitor with a novel chemical structure is a viable strategy for the development of anti-influenza drugs. Structure-based virtual screening methods are an effective tool for the lead identification of novel and promising chemical structures [18,19], with several recent studies illustrating their successful application in drug discovery [20,21]. Here, we report our attempts to find new structural types of PB2 inhibitors using structure-based virtual screening.

## 2. Results

### 2.1. Virtual Screening Strategy

#### 2.1.1. Virtual Screening Strategy Selected

Structure-based virtual screening is an effective strategy for lead compound discovery. At present, there are mainly two types of commonly used structure-based virtual screening methods. One of them is the receptor- or ligand-based pharmacophore searching method [22,23], and the other is the receptor structure-based docking method [24]. The pharmacophore searching method requires fewer computational resources but heavily relies on structure–activity relationship information. Although the docking method requires substantially more computational resources, it can yield better results when there is insufficient information regarding the structure–activity relationship. To date, a number of influenza PB2 protein–active ligand complex crystal structures have been determined; however, the ligand structure diversity is low, and the accumulated structure–activity relationship information is limited. Therefore, we adopted the docking method for virtual screening in our study.

There are a variety of docking methods, and these can be divided into three major subtypes of algorithms: (a) genetic/growing (e.g., FlexX, GOLD, MVP), (b) Monte Carlo-driven (e.g., AutoDock, Flo, Glide, LigandFit), and (c) geometrical- and shape-complementarity-based (e.g., LibDock, Dock4, DockIt, Fred, ICM) [25]. These docking methods use different algorithms, each with its own set of advantages and disadvantages that apply to different scenarios. For example, LibDock is a shape-complementarity-hotspots-based docking method (type C method), which offers a rapid calculation time and is suitable for the screening of large libraries. LigandFit is a Monte Carlo-driven method for accurately docking ligands into protein active sites (type B method). LigandFit consumes a medium level of computing resources and is suitable for the screening of medium-sized libraries in well-defined binding cavities. Another docking method, GOLD (Genetic Optimization for Ligand Docking), is a genetic algorithm-based method for docking flexible ligands into a protein binding site (type a). GOLD requires medium to longer calculation times and is suitable for the screening of small to medium libraries. Here, we applied a cascade of docking methods, LibDock–LigandFit–GOLD, to take advantage of their respective merits, avoid their disadvantages, and obtain good virtual screening results.

#### 2.1.2. Receptor Selection and Treatment

The crystal structure (PDB: 4P1U) of the influenza A PB2 cap-binding domain (CBD) in a complex with VX-787, which has a good protein integrity and a relatively high resolution (2.52 Å), was selected as the receptor for docking studies. The protein was processed with the Prepare Protein protocol in Discovery Studio 3.0 (DS 3.0) to resolve some of the missing atoms, add the hydrogen atoms, and minimize the energy, so as to standardize the protein for subsequent calculations.

#### 2.1.3. Verification of Docking Method

To investigate the applicability and reliability of the docking method, we first attempted to redock the ligand VX-787 to its binding site in the crystal structure using each of the LibDock, LigandFit, and GOLD docking methods. The binding site, docking parameters, and scoring function we adopted are listed in Table S1. During the parameter testing process with GOLD, we found that two key water molecules, HOH 141 and HOH 146, mediated a hydrogen-bonding network between the receptor and VX-787 [26]. This network had a marked impact on obtaining a pose closer to that of the real pose, so these two water molecules were retained in the protein and allowed to toggle and spin. In contrast, these two water molecules had little effect on whether we obtained the expected ligand pose when applying LibDock and LigandFit. Therefore, these water molecules were not taken into consideration when using the latter two methods. After performing the redocking experiments, the predicted highest-ranking poses were compared with the original pose (Figure S1), and the root-mean-square deviation (RMSD) value for each pair of poses was calculated to evaluate the results (Table S1). All the RMSD values were below 1.0 Å, suggesting a reasonable performance of these docking protocols.

#### 2.1.4. Virtual Screening and Molecular Docking

**Figure 1:**
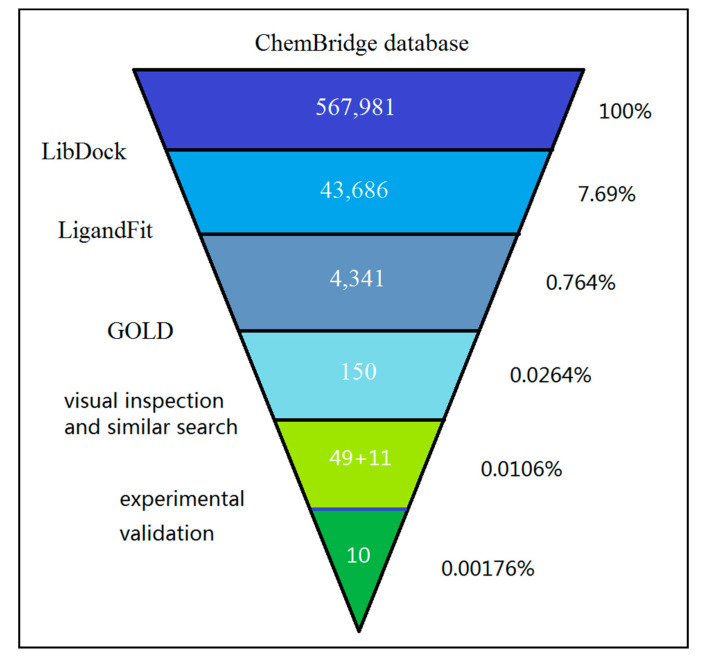
Workflow of the cascade docking virtual screening of ChemBridge database targeting influenza PB2 CBD.

A schematic workflow of the cascade docking virtual screening approach is presented in Figure 1.

A ChemBridge database consisting of 567,981 molecules was prepared and then used to generate the corresponding 3D conformations in DS 3.0. The prepared 3D conformation database was screened using cascade docking with LibDock–LigandFit–GOLD, and the binding site, key docking parameters, and scoring function used by these protocols were consistent with those used during docking method verification. These results are described in Table S1.

First, the database was screened using the LibDock protocol to quickly filter out inappropriate compounds. Rigid ligand docking was performed using the fast search model, and docking was refined using the Smart Minimizer Broyden–Fletcher–Goldfarb–Shanno (BFGS) optimization algorithm. The refined poses were scored with the LibDock scoring function, and the top-scoring pose of each molecule was selected and ranked. Analysis of the high-scoring compounds revealed that a large proportion of them had relatively large molecular weights. The average molecular weight of the top 10% LibDock-scoring compounds was approximately 418. To select the high-scoring compounds with a small molecular weight (as low molecular weight molecules have more optimization space), the ligand efficiency LEMW (LibDockScore/Molecular Weight) of each virtually screened compound was calculated. The top 33% LibDockScore compounds among the top 33% LEMW compounds (43,686 compounds) were selected to go to the next round of screening. The average molecular weight of these hits was reduced to approximately 360.

Next, the selected 43,686 molecules were further screened using the LigandFit protocol (Figure 1). LigandFit searches for poses consistent with the binding site shape using an algorithm that combines a shape comparison filter and a Monte Carlo conformational search method. An internal scoring function, the DOCK SCORE, was used to estimate the binding affinity of ligands to the receptor. Only the highest DOCK SCORE pose was reserved for sorting [27,28]. A final 4341 ligands with a DOCK SCORE >80 (approximately 10% of the scored compounds) were retained for further screening (Figure 1).

These 4341 compounds were then submitted to GOLD v4.1. Docking calculations were performed using the default GOLD fitness function and default evolutionary parameters. Two water molecules (HOH 141, HOH 146) were assigned a versatile state (toggle and spin) to account for their effects. The interacting ability of each compound was ranked based on the GOLD Score. Only the best solution for each ligand participated in the sorting. The top 150 compounds retained based on the GOLD Score entered visual inspection (Figure 1). They were clustered into 10 clusters using the Clustering Molecules protocol in DS 3.0 based on the FCFP_6 fingerprints for visual inspection. The selection criteria for visual inspection were the following: (1) the molecules bound deep inside the PB2 CBD and were a reasonable fit without high strain energy; (2) for those molecules with the same scaffold, the molecules with high ligand efficiency were selected. At last, 49 candidates were selected and purchased for biological evaluation (Figure 1). The structure, docking scores, ID number, and internal serial number of each of these compounds are shown in Figure S2A, Figure S2B, Figure S2C and Figure S2D.

### 2.2. Biochemical Assays

**Figure 2:**
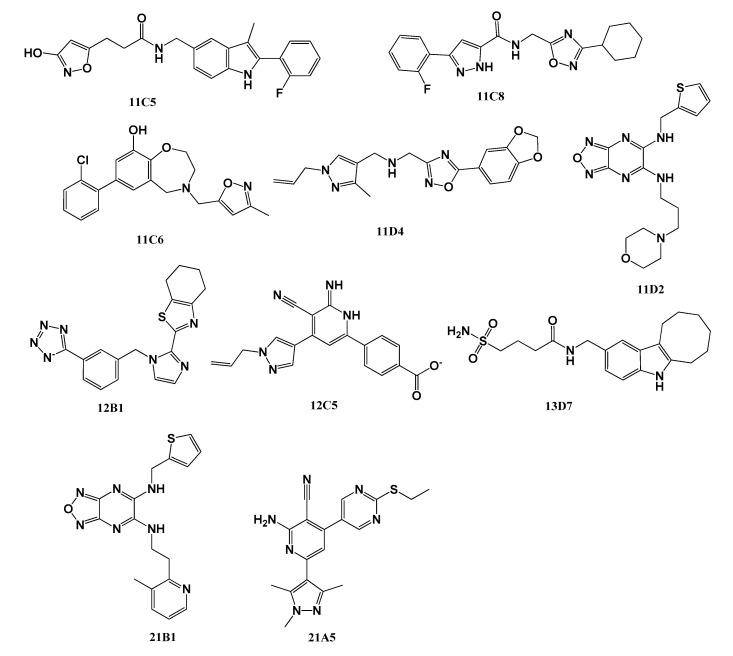
The structures of the 10 experimentally validated compounds.

First, the antiviral activities of the candidate compounds against the influenza A/Puerto Rico/8/1934 (PR/8, H1N1) virus were determined in Madin–Darby canine kidney (MDCK) cells with cytopathic effect (CPE) inhibition assays. Oseltamivir carboxylate (OC) and VX-787 were used as the positive controls. Eight compounds (11C5, 11C6, 11C8, 11D2, 11D4, 12B1, 12C5, and 13D7) with novel scaffolds were found to rescue cells from virus-induced cytopathic effects at non-cytotoxic concentrations (Figure 2). The EC_50_ values of these compounds ranged from 0.30 to 55.50 μM (Table 1). The EC_50_ value of the most active compound, 11D-2, was 0.30 ± 0.02 μM, which was much higher than that of VX-787 (<0.005 μM) but superior to that of OC (1.78 ± 0.72 μM). To expand the range of active compounds, compounds structurally similar to 11D2, 11D4, and 12C5 were searched in the ChemBridge database and virtually screened, and 11 compounds were selected for activity evaluation (Figure 1 and Figure S2). Another two compounds, 21B1 and 21A5, were found to rescue cells from virus-induced cytopathic effects at non-cytotoxic concentrations (Figure 2 and Table 1). Further evaluation showed that four compounds, namely, 21B1, 11D4, 12C5, and 21A5, had a broad-spectrum antiviral effect on various subtypes of influenza viruses. Except for compound 21B1 showing no activity against the A/Hong Kong/8/68 (HK/68, H3N2) strain, these compounds were active against a variety of influenza A virus strains, including HK/68 (H3N2), A/WSN/33 (H1N1), A/LiaoNing-ZhenXing/1109/2010 (ZX/1109, H1N1, natural isolate oseltamivir-resistant), the PR/8-R292K mutant (H1N1, recombinant oseltamivir-resistant), the PR/8-I38T mutant (H1N1, recombinant baloxavir-resistant), and influenza B/Lee/40 virus strains (Table 1). The EC_50_ of these compounds ranged from 0.30 to 67.65 μM. It was not surprising that the oseltamivir- and baloxavir-resistant strains were sensitive to our compounds, because our compounds have different targets other than oseltamivir and baloxavir, and thus mutations of amino acid residues in the target sites of oseltamivir and baloxavir binding have no effect on these compounds. However, our compounds had similar inhibitory effects on various wild strains of influenza A and influenza B, which was unexpected, because the sequence identity between influenza A and influenza B is only around 34% (Figure S3). In addition, according to literature reports, the activity of VX-787 against influenza A viruses and influenza B viruses differs greatly [26]. The cytotoxicity of these compounds was determined in MDCK cells, and the cell cytotoxicity (CC_50_) observed for all 10 compounds was >100 µM (Table 2).

To confirm that these 10 active compounds are competitive ligands of PB2 CBD, biomolecular interaction analysis based on a surface plasmon resonance (SPR) competitive binding assay was conducted. Consistent with expectations based on previous molecular docking experiments, moderate binding affinities with equilibrium dissociation constants (*K_d_*) ranging from 0.5 to 6.7 μM were found between these compounds and influenza PB2 CBD (Table 1). Compounds 11D2 and 21A5 exhibited a concentration-dependent association–dissociation pattern with a *K_d_* of 0.21 and 0.54 μM, respectively. There was approximately an order of magnitude difference in the *K_d_* compared with that of the control compound, VX-787 (0.054 μM).

In short, through a virtual screening and similarity search, we selected a total of 60 compounds for antiviral activity evaluation. The acceptance rate of virtual screening is about 0.0106% (Figure 1). Ten compounds were found to have obvious inhibitory effects on the H1N1 influenza virus, accounting for 16.7% of the 60 tested compounds, with 4 of these compounds exerting broad-spectrum antiviral effects on multiple influenza strains.

## 3. Discussion

**Figure 3:**
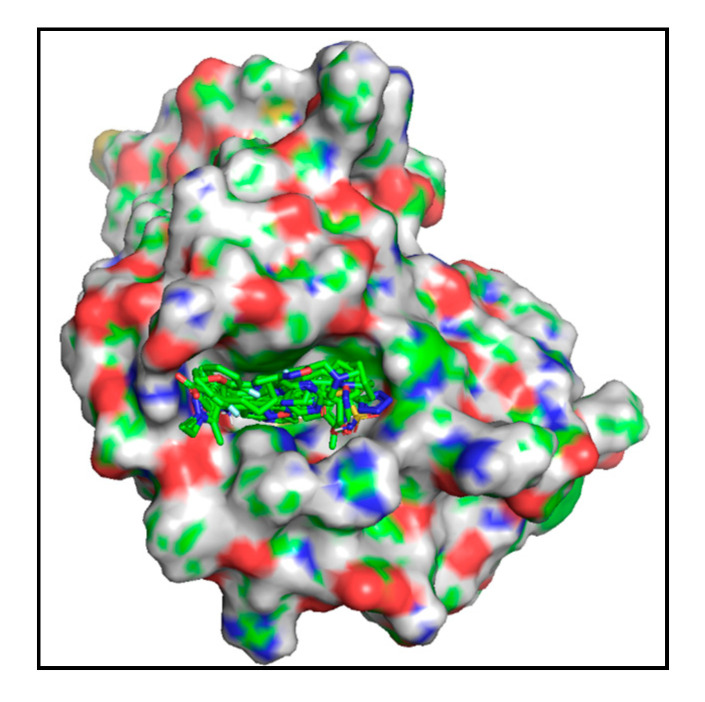
The protein complexes of PB2 CBD with active compounds. The active compounds are represented with colored stick models.

In short, through a virtual screening and similarity search, we selected a total of 60 compounds for antiviral activity evaluation. Thereupon, 10 compounds were found to possess an obvious antiviral effect on H1N1 (PR/8), and 4 of these offer broad-spectrum antiviral effects on multiple subtypes of influenza virus, including influenza virus B, with no significant cytotoxicity. The SPR competitive binding assay indicated that these compounds show good binding to the influenza virus PB2 CBD. Because the influenza virus PB2 CBD site has no other obvious binding sites (Figure 3), these compounds can be confirmed to bind to the influenza virus PB2 CBD site.

### 3.1. Binding Models Analysis of Active Compounds to PB2 CBD

The X-ray crystal structure of VX-787 bound to PB2 (PDB: 4P1U) confirmed that VX-787 and PB2 are tightly bound. The azaindole ring system interacts with the protein residues Lys376 and Glu361, and the aromatic rings of VX-787 stack between the side chains of His357, Phe323, Phe363, and Phe404 to form a typical sandwich structure with His357 and Phe404. The carboxylic group of the ligand participates in two water-mediated interactions with the ε nitrogen of His357 and Gln406, as well as the main chain carbonyl of Arg355. Additional salt bridge interactions with Arg355 in the adjacent region were also observed [17]. Interaction fingerprints analysis showed that the allylpyrazole occupied the hydrophobic cavity formed by residues Phe323, Phe325, Phe404, and Met431. In addition, the methylenedioxybenzene fragment interacts with Phe323 and Phe363 through hydrophobicity (Figure S3). This is consistent with the high affinity of VX-787 toward PB2 and its significant antiviral activity.

**Figure 4:**
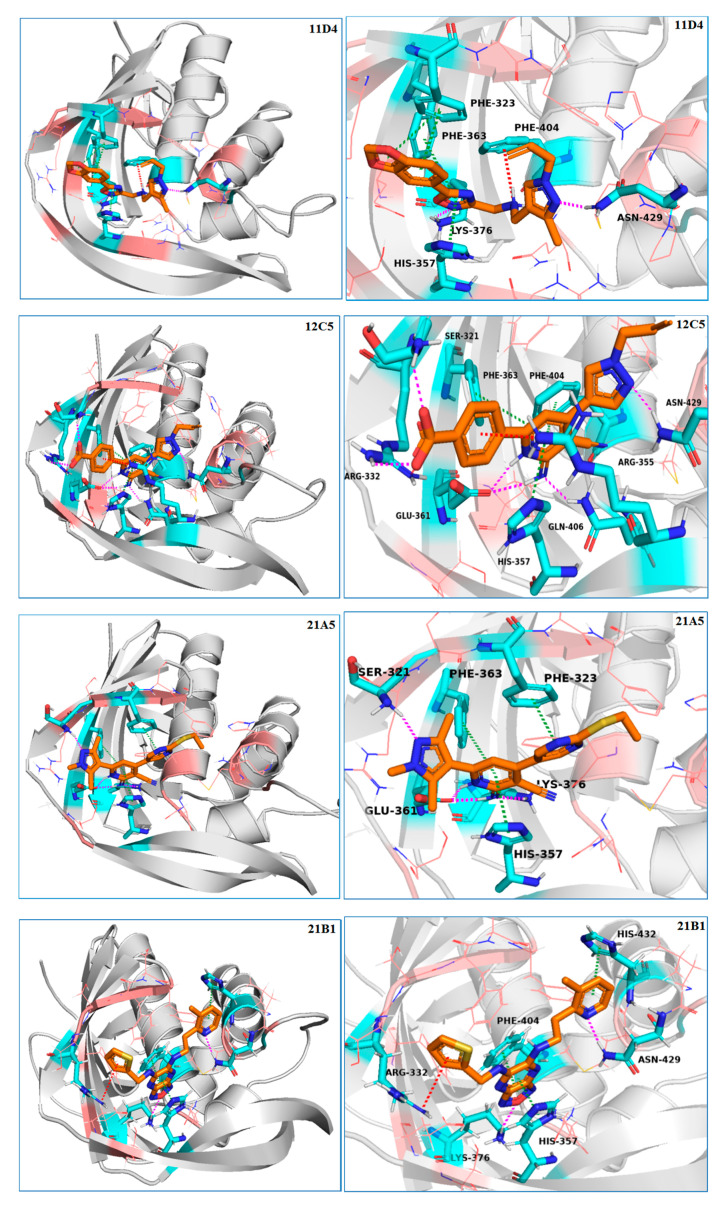

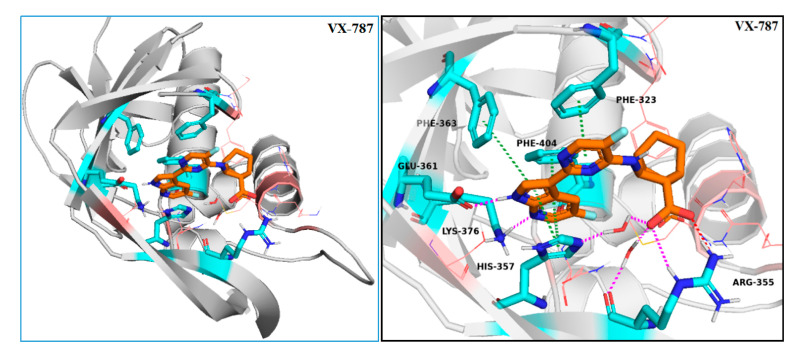
The molecular docking models of the cap-binding domain (CBD) of the PB2 complex with compounds 11D4, 12C5, 21A5, and 21B1. The CBD of PB2 derived from PDB 4P1U is represented with a gray cartoon. The ligands are shown as orange sticks; the amino acid residues within 4 Å of the ligand that participate in hydrogen bonds, salt bridges, π–π stacking, and cation–π stacking with the ligand are shown as cyan sticks; and other amino acid residues within 4 Å of the ligand are shown in pink. Hydrogen bonds are represented by dashed magenta lines, π–π stacking is represented by dashed forest-green lines, and salt bridges and cation–π stacking are represented by dashed red lines.

Our 10 experimentally validated compounds were significantly different in structure from VX-787. From the analysis of the predicted optimal binding modes of these compounds obtained from the docking results, we found that our active molecules bound deep within the PB2 CBD, similar to VX-787 and without high strain energy (Figure 3). To investigate the binding models of our active compounds in detail, the docking models of four representative compounds, 11D4, 12C5, 21A5, and 21B1, were optimized using the Refine Protein–Ligand Complex tool of Prime. The results showed that the binding modes of these compounds were somewhat similar to that of VX-787, but there were also many unique features (Figure 3, Figure 4 and Figure S6). First, similar to VX-787, compounds 12C5, 11D4, 21A5, and 21B1 all exhibited π–π stacking between their aromatic ring and the imidazole ring of His357. At the same time, these compounds were partially involved in π–π stacking interactions with the side-chain phenyl rings of Phe363, Phe323, and Phe404. Second, another similarity to VX-787 is that all four active compounds maintained hydrogen bonding with Lys376, whereas compound 12C5 simultaneously maintained the hydrogen bond with Glu361. Third, interaction fingerprint analysis showed that our compounds also retained the hydrophobic effects of the side chains of Phe323, Phe325, Met431, Phe404, and Phe363, similar to that of VX-787 (Figure S3).

Since our compounds are structurally distinct from VX-787 even though they also bind to the CBD site, they exhibited several completely different additional protein-binding modes from that of VX-787. First of all, our compounds had a different π–π stacking effect and cationic–π interactions from those of VX-787. Compound 11D4 had no π–π stacking with the side-chain benzene ring of Phe404 as VX-787 did, while a cationic–π interaction was observed between Phe404 and the amino cation of compound 11D4. For compound 21B1, no π–π stacking with the side-chain benzene ring of Phe323 and Phe363 was observed, while there was a cationic–π interaction between the guanidino group of Arg332 and the thiophene ring, and π–π stacking between the imidazole of His432 and picoline was also found. In addition, the hydrogen bond between our compound and PB2 was markedly different from that of VX-787. For compound 21A5, the pyrazole formed a hydrogen bond with the side chain of Ser321. For compound 12C5, the carboxyl group formed two H-bonds with Arg332 and Ser321. The unsubstituted nitrogen atom of allylpyrazole formed a hydrogen bond with the amide hydrogen atom of Asn429 (Figure 4). Similar to compound 12C5, compounds 11D4 and 21B1 maintained hydrogen bonding with Asn429. Besides, the carboxyl group of 12C5 also formed a salt bridge with the protonated guanidino group of the Arg332 side chain, which is absent in VX-787. One of the most significant differences between our compounds and VX-787 is that our compounds lack an acidic group to form a salt bridge with Arg355, and they do not interact with His357 and Gln406 via water molecules.

There is still a large gap between the anti-influenza activities of the compounds we obtained and that of VX-787. This is because the structures have not been optimized; thus, the compatibility of these compounds as ligands of PB2 is still far from that of VX-787. First, the azaindole ring of VX-787 forms a typical sandwich structure with His357 and Phe404, but none of our compounds (except 21B1) can form such a perfect sandwich structure. Second, the bicyclo[2.2.2]octane of VX-787 is a large hydrophobic group that has considerable contact with the hydrophobic cavity of PB2, while none of our compounds have a sufficiently large hydrophobic group. Third, our compounds have no acidic group and can neither form salt bridges and hydrogen bonds with Arg355 guanidine nor form hydrogen bond networks with His357 and Gln406 through water molecules.

Although the antiviral activities of our compound are still far behind that of VX-787, our compounds have novel chemical scaffolds and relatively small molecular weights and, thus, are suitable for optimization as lead compounds. By optimizing and modifying our compounds, such as through adding aromatic rings or adjusting the position of the aromatic rings to increase or strengthen the π–π stacking interactions, and introducing large hydrophobic groups and an acidic group to interact with the Arg355 guanidine, we believe that it will result in more active compounds.

### 3.2. Influenza PB2 CBD Structure and Sequence Alignment and Compound Selectivity Analysis

It is reported in the literature that VX-787 has a significant inhibitory effect on influenza B virus 23. In contrast, our compounds, 11D4, 12C5, 21A5, and 21B1, had similar inhibitory effects on various strains of influenza A and influenza B. To explore the reasons why our compounds exhibited different selectivity toward influenza A and influenza B compared with VX-787, we compared the sequences and structures of the PB2 CBD site of several wild strains of influenza. The protein sequence of PDB 4P1U (PB2 CBD of A/Victoria/3/1975 (H3N2) bound to VX-787) was directly extracted from the PDB file. The sequences of PB2 of the human influenza A/WSN/1933 (H1N1), ZX/1109 (H1N1), A/Hong Kong/16/1968 (H3N2), and B/Lee/40 virus strains were downloaded from the Influenza Virus Resource (http://www.ncbi.nlm.nih.gov/genomes/FLU/FLU.html) and Global Initiative on Sharing Avian Influenza Data (http://platform.gisaid.org/) databases. These protein sequences were aligned, and the similarity between the amino acid sequences in the alignments was calculated using the Align Sequence protocol in DS 3.0 (Figure S5). The identity between the PB2 CBD sequence of the A/Victoria/3/1975 (H3N2) virus and that of the A/Hong Kong/16/1968 (H3N2) virus reached 97.5%. The identity between the PB2 CBD sequence of A/Victoria/3/1975 (H3N2) and that of ZX1109 (H1N1) and A/WSN/1933 (H1N1) was also high at 92.0% and 94.5%, respectively. This suggests that the PB2 CBD is a highly conserved site in the influenza A virus. In particular, some key amino acid residues, such as Ser321, Phe323, Phe325, Arg332, His357, Glu361, Phe363, Lys376, Phe404, Gln406, Asn429, and Met432, are highly conserved (Figure S5). This fact may well explain the broad-spectrum antiviral activity of our compounds and VX-787 against various influenza A viruses.

**Figure 5:**
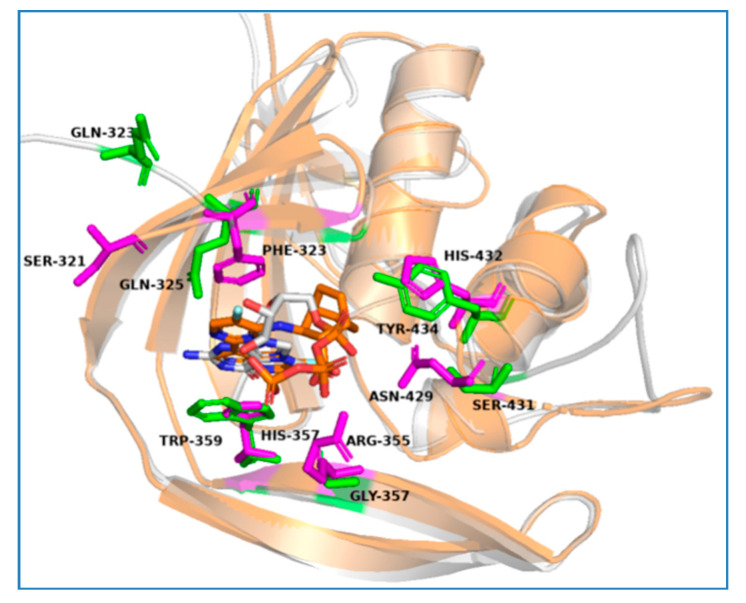
Three-dimensional structural overlay of the PB2 CBD of influenza A (H3N2) (PDB 4P1U, bound to VX-787) and influenza B/Lee/40 (PDB 5EFA, bound to M7GTP) and the key variant amino acid residues. PDB 4P1U is represented as an orange cartoon, and its key amino acid residues are shown with magenta sticks. PDB 5EFA is represented as a gray cartoon, and its key amino acid residues are shown with green sticks.

In contrast, the PB2 CB sequence identity between the A/Victoria/3/1975 (H3N2) and the B/Lee/40 virus strains is only 35.0%, indicating that the PB2 CBD sequences of influenza A and influenza B are significantly different (Figure S4). The 3D structure overlay results of PDB 4P1U and PDB 5EFA (B/Lee/40 PB2 CBD bound to M7GTP) showed that their structural similarity is higher than their sequence similarity (Figure 5). Analysis of these two protein sequences showed that several key amino acid residues, such as Phe325/327, Arg332/334, Glu361/363, Phe363/365, Lys376/378, Phe404/406, Gln406/408, and Asn429/431 (amino acid residue numbers of PDB 4P1U/PDB 5EFA, respectively), are conserved in both type A and type B influenza viruses (Figure S5). However, some key amino acids, including Phe323/Gln325, His357/Trp359, His432/Tyr434, Asn429/Ser431, and Arg355/Gly357, are mutated. Compounds 21B1, 11D4, 12C5, 21A5, and VX-787 participate in π–π stacking with His357. Careful observation suggests that the His357/Trp359 mutation would not have an adverse effect on the activity of PB2 CBD inhibitors, because the indole ring system of Trp residues can also form a π–π stack similar to the imidazole ring of His, and as the indole ring system is larger than the imidazole ring, this π–π stack should be enhanced. On the other hand, the mutation from Phe323 to Gln325 would destroy a key π–π stack; hence, this mutation should have an adverse effect on the activities of compounds that directly interact with it, including VX-787, 21B1, 11D4, and 21A5. However, the experimental results indicate that this variation does not exert much effect on the inhibitory activities of these compounds toward influenza A and influenza B. This may be because the loss of binding energy caused by this mutation is compensated to a certain extent by the enhancement of the π–π stack effect by the His357/Trp359 mutation. The antiviral activities of 11D4, 12C5, 21A5, 21B1, and VX-787 should be little affected by the His432/Tyr434 mutation, for only compound 21B1 has a direct interaction (π–π stacking) with this residue, and the mutated Tyr434 can maintain a similar interaction. There is no direct interaction between VX-787 and Asn429, so the Asn429/Ser431 mutation should have little effect on the activity of VX-787. Among our compounds, 11D4, 12C5, and 21B1 have hydrogen bonds with Asn429. However, the experimental results show that this variation does not have much effect on the inhibitory activities of these compounds on influenza A and influenza B. The Arg355/Gly327 mutation should be key, because the carboxyl group that interacts directly with this amino acid residue plays a key role in maintaining VX-787 activity [17], and this mutation would destroy a key salt bridge and two hydrogen bonds (Figure 2, Figure 3 and Figure 4). None of our compounds contain an acid group that can interact with Arg355 as VX-787 does, and this may be the most critical reason why our compounds are not sensitive to the PB2 CBD sequence variation of influenza A and influenza B and have similar inhibitory activities toward both. At the same time, this may be a key reason why the anti-influenza virus activities of our compounds are inferior to that of VX-787.

Based on the above analysis and visual observation of Figure 5, the three residue mutations that have a greater impact on the binding abilities of influenza PB2 CBD inhibitors should be Phe323/Gln325, Asn429/Ser431, and Arg355/Gly357. When attempting to obtain compounds with high activities against both influenza A and influenza B, it is critical that the impact of these mutations be considered fully, particularly the impact of Arg355/Gly357.

## 4. Materials and Methods

### 4.1. Virtual Screening

#### 4.1.1. Protein Preparation

The crystal structure of influenza A PB2 complexed with VX-787 was retrieved from the Protein Data Bank (PDB code: 4P1U) and further processed with the Prepare Protein module in Discovery Studio 3.0 (DS 3.0). A missing loop was added, the incomplete side chains of residues Glu341 and Arg436 were corrected, the bond orders were corrected, and the protonation states of the residues were adjusted to the dominant ionic forms at pH 7.4. The hydrogen atoms were added, and their positions were optimized using the all-atom CHARMM force field with the adopted basis NR minimization algorithm, until the root mean square (RMS) gradient for potential energy was <0.05 kcal/mol Å.

#### 4.1.2. Ligand Setup

Freely accessible SDF files from the ChemBridge databases comprising a total of 384,796 compounds were obtained. The ligands were prepared using the Prepare Ligands protocol in DS 3.0 with default parameters, generating isomers and tautomers. The three-dimensional (3D) conformation library of these isomers and tautomers was generated using the Generate Conformations protocol in DS 3.0 with the fast method to facilitate the LibDock work in the subsequent step.

#### 4.1.3. Virtual Screening Using LibDock

LibDock was used to perform high-throughput screening of the generated ChemBridge compound 3D conformation library with the fast search model. The binding site was defined as the volume occupied by VX-787, and an input site sphere (−48.6597, −5.33945, 4.06342, 9) defined over the binding site was selected for molecular docking analysis. The number of hotspots was set to 100. The receptor protein conformation was kept fixed during docking, and the docked poses were further minimized using the CHARMM force field and the smart minimization method, until the RMS gradient for potential energy was <0.001 kcal/(mol Å). The atoms of the ligand and the side chains of the residues of the receptor within the binding site sphere were kept flexible during minimization. The docked pose with the highest LibDock score was retained for each compound. The LEMW (LibDockScore/Molecular Weight) of each retained compound was calculated. The top 33% LibDock-scoring compounds among the top 33% LEMW compounds (37,686 compounds) were selected to enter the next round of screening.

#### 4.1.4. Virtual Screening Using LigandFit

The 37,686 compounds obtained by screening with LibDock were further subjected to LigandFit docking. A site with a volume of 393.500 Å^3^ and a point count of 3148 with equal grid spacing in each dimension of 0.5 (X), 0.5 (Y), and 0.5 (Z) was detected with the site detection algorithm implemented in LigandFit, and this was designated as the binding site. LigandFit docking was carried out using the Dreiding parameter. In this energy grid parameter, the Gasteiger charge method was employed to calculate the partial charges of the target protein and ligand. The energy grid extension was set to 3.0 Å, and the conformation search number of the Monte Carlo trial was set to “use the variable number from the given table.” The input parameters for docking were set to the default options, except for the number of poses for ligands in the receptor cavity, which was set to 10. The pose optimization was performed with the Broyden–Fletcher–Goldfarb–Shanno (BFGS) method and evaluated based on the DOCK_SCORE. The docked poses were not further minimized because of time considerations. A total of 4341 ligands with a DOCK_SCORE >80 were retained for further docking.

#### 4.1.5. Virtual Screening Using GOLD

GOLD v4.1 (http://gold.ccdc.cam.ac.uk./index.php) was used for higher-precision molecular docking. The crystal structure of PDB 4P1U was used directly for the GOLD docking process, the hydrogen atoms were added, and all except two water molecules were removed from the protein (HOH 141 and HOH 146). The binding site of the ligand was defined as a 9 Å radius from the bound ligand, VX-787. Docking calculations were performed using the default GOLD fitness function and default evolutionary parameters. The two retained water molecules were set to toggle and spin. Ten docking runs were performed per structure unless 3 of the 10 poses were within 1.5 Å RMSD (root-mean-square deviation) of each other. The GA setting was set to automatic modeling with a search efficiency of 100%. The ligand flexibility parameters were set to default. The interacting ability of a compound depends on the GOLD fitness score; therefore, only the top solution for each ligand participated in the sorting and the top-ranking 150 ligands were selected for further visual inspection.

#### 4.1.6. Refining the Protein–Ligand Complex

The predicted binding modes of the active compounds obtained from our GOLD docking results were further optimized using the Refine Protein–Ligand Complex tool of Prime (Schrodinger Suite 2016). The OPLS3 force field was selected along with the VSGB solvation model. The whole protein–ligand complex was optimized by minimization using the hierarchical optimization algorithm. Fifty clusters were used when clustering the conformations of the complex, and only the optimal structure was returned. No constraints were adopted. The environment options were set to default, the seed option was set to a constant zero, and the dielectric option was set to 80.

### 4.2. Cell Lines and Virus Strains

The Madin–Darby canine kidney (MDCK) cell line was purchased from the American Type Culture Collection (ATCC, Manassas, VA, USA) and maintained at 37 °C in Dulbecco’s modified Eagle’s medium (DMEM) supplemented with 10% fetal bovine serum, 100 μg/mL streptomycin, and 100 U/mL penicillin. Influenza A PR/8 (H1N1), ZX/1109 (H1N1), the PR/8-R292K mutant (H1N1, oseltamivir-resistant, recombinant strain), the PR/8-I38T mutant (H1N1, baloxavir-resistant, recombinant strain), A/WSN/33 (H1N1), HK/68 (H3N2), and influenza B/Lee/40 were propagated in 8- to 10-day-old embryonated chicken eggs or MDCK cells for 3 days at 37 °C. The virus strains were stored in our laboratory.

### 4.3. Cytopathic Effect (CPE) Inhibition Assay

MDCK cells were seeded and grown for 18–24 h to a confluent monolayer in a 96-well plate. The medium was exchanged with DF-12 medium containing 2 μg/mL TPCK-trypsin after washing twice with phosphate-buffered saline (PBS). Cells were infected with each influenza virus strain at a multiplicity of infection (MOI) of 0.005 in DF-12 medium containing 2 μg/mL TPCK-trypsin in the presence of various concentrations (ranging from 0.005 to 100 μM by three-fold dilution) of each test compound. After a 72 h incubation at 37 °C in a CO_2_ incubator, the antiviral activity of the test compounds was measured using the CellTiter-Glo viability assay (Promega, Madison, WI, USA). The concentration required for 50% maximal effect (EC_50_) was calculated using Origin 8 software.

### 4.4. Cytotoxicity Assay

The cytotoxicity of the compounds was evaluated in MDCK cells using the CellTiter-Glo viability assay according to the manufacturer’s instructions. Briefly, cells were seeded at a density of 1.5 × 10^4^ per well into 96-well plates and grown for 18–24 h to a confluent monolayer. The medium was exchanged with DF-12 medium containing 2 μg/mL TPCK-trypsin, and the test compounds were added to the cells in a three-fold dilution series. DMSO was added for the control. After a 72 h incubation at 37 °C in a CO_2_ incubator, the luminescence of each well was read with a SpectraMax M5 microplate reader (Molecular Devices, San Jose, CA, USA). The 50% cytotoxicity concentration (CC_50_) was calculated using Origin 8.

### 4.5. Surface Plasmon Resonance (SPR) Analysis

For H1N1 PB2 CBD protein expression and purification, a 507 bp region of the target gene, PB2 from influenza A/WSN/1933 (H1N1, a.a. 318–486), GenBank ID: BBB04699.1, was amplified from the plasmid, spET24-6H-JS30-2, constructed by Sino Biological, Inc. (Beijing, China), using primers 190822-F2 and 190822-R1. This 507 bp fragment was used as the template to amplify a 531 bp region using primers 190822-F3 and 190822-R1 to add a 3C protease cleavage site. Finally, the 531 bp fragment was cloned into the vector, spET24-SUMOstar, constructed by Sino Biological, Inc., via homologous recombination using the StuI and XhoI restriction sites, in frame with an N-terminal histidine and SUMO tag. The protein was expressed in *Escherichia coli*, and the fusion protein was captured with a Ni-IDA column. The N-terminal tags were removed by cleavage with HRV 3C protease. The digested protein mixtures were further purified with a Ni-IDA column, yielding the purified PB2 CBD protein of influenza A/WSN/1933 (H1N1, a.a. 318–486). All primer sequences are listed in the supporting information (Table S2).

H1N1 PB2 was immobilized to a CM5 sensor chip (GE Healthcare, Chicago, IL, USA) to a level of approximately 4900 response units (RUs) using a Biacore T200 (GE Healthcare) and a running buffer composed of 1× PBS-P buffer (PBS, pH 7.4, containing 0.02 M phosphate buffer, 2.7 mM KCl, 0.137 M NaCl, and 0.05% Tween 20). Serial dilutions of the compounds (VX-787, 11C5, 11C6, 11D2, 11D4, 11C8, 12B1, 12C5, 13D7, 21A5, and 21B1) were injected at concentrations ranging from 20 to 0.156 µM. The resulting data were fit to the affinity binding model using the Biacore Insight Evaluation Software 2.0.2 (GE Healthcare).

## 5. Conclusions

Influenza treatment remains an unresolved clinical need. Targeting the influenza virus RNA polymerase PB2 subunit is considered an ideal strategy for the development of anti-influenza drugs. However, existing PB2 inhibitors are not ideal. In this study, we carried out virtual screening based on LibDock–LigandFit–GOLD cascade docking to discover new inhibitors of the PB2 cap-binding domain. Among the 567,981 compounds screened, 60 candidates were selected for testing by plaque reduction assays and surface plasmon resonance assay. Ten compounds with novel scaffolds were identified to rescue macrophages from H1N1 virus-mediated death at non-cytotoxic concentrations with EC_50_ values ranging from 0.30 to 67.65 μM. Among these, four compounds, namely, compound 11D4, 12C5, 21A5, and 21B1, could inhibit a broad spectrum of influenza virus strains, including HK/68 (H3N2), A/WSN/33 (H1N1), ZX/1109 (H1N1, natural isolate, oseltamivir-resistant), the PR/8-R292K mutant (H1N1, recombinant oseltamivir-resistant strain), and the influenza B/Lee/40 virus. An SPR protein binding affinity assay indicated that these compounds could bind to PB2 CBD at low micromolar concentrations. Although the antiviral activities of our compounds are still far behind that of VX-787, our compounds offer novel chemical scaffolds and relatively small molecular weights and, thus, are suitable for optimization as lead compounds.

Based on the sequence alignment of PB2 CBD sites of different subtypes of influenza viruses and the 3D structural alignment of PDB 4P1U and PDB 5EFA (B/Lee/40 PB2 CBD bound to M7GTP), we suggest that the Phe323/Gln325, Asn429/Ser431, and Arg355/Gly357 mutations, particularly the Arg355/Gly357 mutation, will have a major impact on the selectivity of PB2 CBD inhibitors of influenza A and influenza B. To obtain compounds with high activity against both influenza A and influenza B, the impact of these mutations needs to be fully considered.

## Figures and Tables

**Table 1:** The structure, docking scores, anti-H1N1-PR/8 activities, cytotoxicity, and *K_d_* of the active compounds.

Compounds	LibDock (LibDock Score)	LigandFit (DOCK SCORE)	GOLD (Gold Score Fitness)	Anti-PR/8 (H1N1), Activities (EC_50,_ μM)	Cytotoxicity (CC_50,_ μM)	*Kd* for H1N1 PB2 (μM)
11C5	135.11	160.86	84.78	55.50 ± 27.66	>100	2.26
11C6	123.98	85.29	58.99	35.61 ± 1.74	>100	1.70
11C8	129.66	85.38	74.95	26.38 ± 12.04	>100	6.16
11D2	130.29	91.66	77.89	0.30 ± 0.02	>100	0.21
11D4	132.49	82.76	65.66	6.20 ± 2.85	>100	1.87
12B1	136.28	156.10	68.13	53.92 ± 10.36	>100	6.77
12C5	125.19	154.16	76.52	30.88 ± 2.58	>100	2.44
13D7	132.24	86.26	73.07	26.33 ± 13.84	>100	1.13
21B1	132.55	96.99	75.31	67.65 ± 43.28	>100	1.95
21A5	115.88	89.57	65.54	22.07 ± 10.99	>100	0.54
VX-787				<0.005	>100	0.054
OC				1.78 ± 0.72	>100	

**Table 2:** The activities of the compounds against multiple influenza virus strains.

Cp.	Cytotoxicity	HK/68	A/WSN/33	B/Lee/40	PR/8-R292K *	ZX/1109 *	PR/8-I38T #
	CC_50_ (μM)	EC_50_ (μM)	EC _50_ (μM)	EC _50_ (μM)	EC _50_ (μM)	EC _50_ (μM)	EC_50_ (μM)
11D4	>100	25.81 ± 1.89	19.25 ± 6.28	11.29 ± 0.15	21.47 ± 5.49	38.37 ± 3.49	32.44 ± 1.52
12C5	>100	30.10 ± 12.76	24.11 ± 6.14	25.12 ± 2.57	28.61 ± 0.99	36.66 ± 2.33	35.42 ± 1.02
21B1	>100	>100	57.43 ± 15.46	60.35 ± 1.41	14.61 ± 1.44	11.83 ± 1.07	37.01 ± 0.67
21A5	>100	25.62 ± 18.79	14.79 ± 6.31	33.81 ± 0.68	9.27 ± 0.55	5.71 ± 1.14	14.14 ± 0.47
VX-787	>100	1.84 ± 0.19	ND	>10	4.14 ± 0.66	2.25 ± 0.05	2.34 ± 0.10
Oseltamivir carboxylate	>100	0.004 ± 0.001	0.09 ± 0.02	4.04 ± 0.58	>100	>100	ND

* Oseltamivir-resistant strain, # baloxavir-resistant strain. ND, not done.

## Supplementary Materials

The following are available online. Figure S1: Comparison between the highest-ranked pose of VX-787 and the original pose in the crystal structure, Figure S2: The structure, docking scores, ID, and internal serial number of obtained compounds by virtual screening, Figure S3: The hydrophobic amino acid residues interacting with the ligand found by interaction fingerprints analysis and the hydrophobic interaction diagram, Figure S4: PB2 cap-binding domain sequence alignment of influenza A/Victor/3/1975 (H3N2) and influenza B Lee, Figure S5: The sequence alignment results of the CBD site of influenza A virus and influenza B (partial) and the key amino acids that interact with active compounds. Table S2: The sequences of the primers.
