# Supplementary material for: Identification of Novel Influenza Polymerase PB2 Inhibitors Using a Cascade Docking Virtual Screening Approach
Source: Molecules. 2020 Nov 13;25(22):5291. doi: 10.3390/molecules25225291 (PMC7697191; doi:10.3390/molecules25225291)
Supplement: Supplementary file 1 [file molecules-25-05291-s001.pdf]

Surporting information

Table S1. The key docking parameters and RMSD of the predicted highest-ranked pose relative to the original pose in the crystal structure

Dock method	Binding site, Key docking parameters and Score function	RMSD (Å)
LibDock	Binding site: -48.6597, -5.33945, 4.06342, 9; Number of Hotspots:100; Docking Tolerance: 0.25; Docking Preferences: Fast Search; Minimization Algorithm: Smart Minimizer; Score function: LibDockScore.	0.65
LigandFit	Binding site: site1: 3148 points, 393.500 Å^3, partition level 1; energy grid: dreiding; Number of Monte Carlo Trials: "2 500 120, 4 1200 300,6 1500 350,10 2000 500,25 3000 750"; Minimization Algorithm: Smart Minimizer; Minimization Forcefield:CHARMm; Scoring Functions: DOCK SCORE.	0.61
GOLD	Binding site: all atoms within 9Å of VX-787; Water configure: HOH141 and HOH146, toggle and spin; GA settings: Automatic; search efficiency: 100%; Scoring Functions: Gold Score.	0.34

Figure S1. Comparison between the highest ranked pose of VX-787 and the original pose in the crystal structure (Upper left: Libdock docking result comparison; Upper right: Ligandfit docking result comparison; Bottom: Gold docking result comparison.) The original pose of VX-787 is presented as green orange sticks, the docking poses of VX-787 are presented as orange sticks.

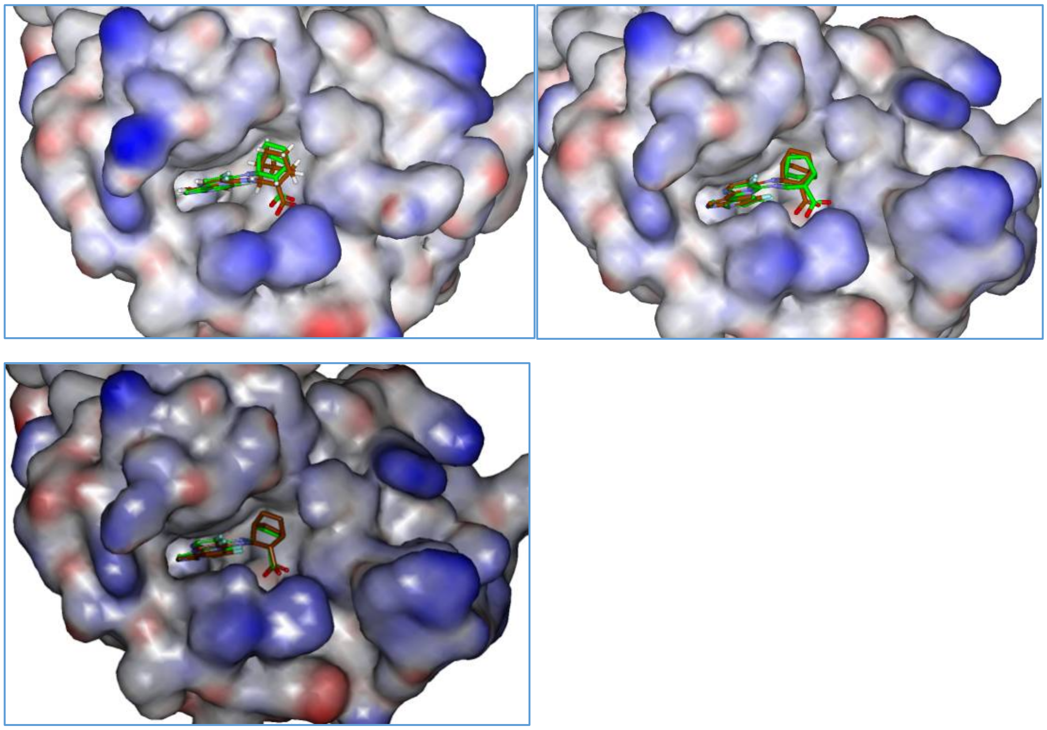

Figure S2. The structure, docking scores, ID and internal serial number of obtained by virtual screening

Figure S2-A

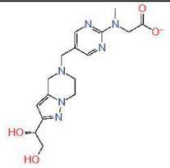<br>ID: 10999800<br>LibDockScore: 102.748<br>DOCK SCORE: 120.7<br>Gold.Goldscore.Fitness: 78.571<br>internal serial number: 11A1	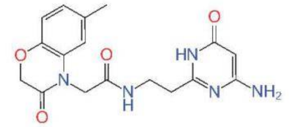<br>ID: 11828541<br>LibDockScore: 118.404<br>DOCK SCORE: 145.772<br>Gold.Goldscore.Fitness: 80.109<br>internal serial number: 11A7	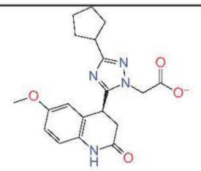<br>ID: 11971416<br>LibDockScore: 116.673<br>DOCK SCORE: 105.351<br>Gold.Goldscore.Fitness: 80.97<br>internal serial number: 13A5
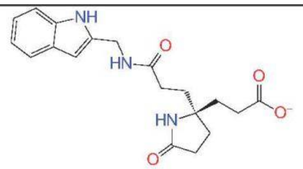<br>ID: 12006803<br>LibDockScore: 125.36<br>DOCK SCORE: 124.796<br>Gold.Goldscore.Fitness: 80.492<br>internal serial number: 13C4	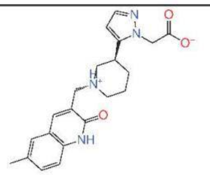<br>ID: 12503475<br>LibDockScore: 142.92<br>DOCK SCORE: 134.327<br>Gold.Goldscore.Fitness: 86.925<br>internal serial number: 11A8	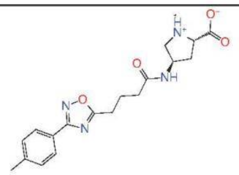<br>ID: 14249086<br>LibDockScore: 110.977<br>DOCK SCORE: 92.387<br>Gold.Goldscore.Fitness: 78.185<br>internal serial number: 11B8
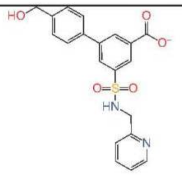<br>ID: 14277921<br>LibDockScore: 132.894<br>DOCK SCORE: 161.987<br>Gold.Goldscore.Fitness: 78.169<br>internal serial number: 11A4	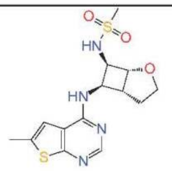<br>ID: 16373629<br>LibDockScore: 126.194<br>DOCK SCORE: 122.605<br>Gold.Goldscore.Fitness: 86.359<br>internal serial number: 12A1	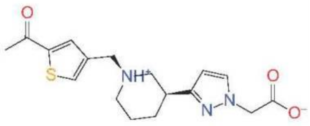<br>ID: 25456024<br>LibDockScore: 103.126<br>DOCK SCORE: 129.43<br>Gold.Goldscore.Fitness: 79.057<br>internal serial number: 12B3
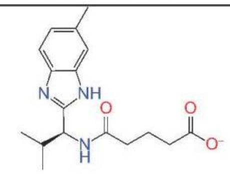<br>ID: 28362130<br>LibDockScore: 121.79<br>DOCK SCORE: 118.761<br>Gold.Goldscore.Fitness: 86.603<br>internal serial number: 13B7	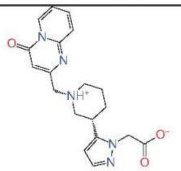<br>ID: 29219691<br>LibDockScore: 125.455<br>DOCK SCORE: 108.73<br>Gold.Goldscore.Fitness: 78.362<br>internal serial number: 11C7	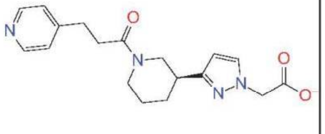<br>ID: 30156393<br>LibDockScore: 106.04<br>DOCK SCORE: 125.902<br>Gold.Goldscore.Fitness: 77.584<br>internal serial number: 13D5
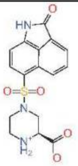<br>ID: 30689910<br>LibDockScore: 124.066<br>DOCK SCORE: 144.001<br>Gold.Goldscore.Fitness: 81.126<br>internal serial number: 11B1	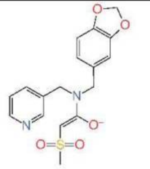<br>ID: 31074889<br>LibDockScore: 109.19<br>DOCK SCORE: 99.335<br>Gold.Goldscore.Fitness: 77.545<br>internal serial number: 11D3	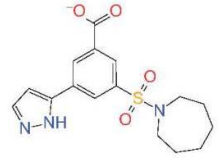<br>ID: 31405729<br>LibDockScore: 111.753<br>DOCK SCORE: 121.152<br>Gold.Goldscore.Fitness: 78.068<br>internal serial number: 12D5

Figure S2-B

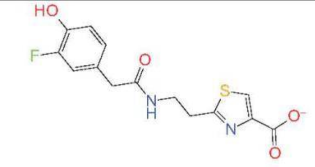

ID: 32053166
LibDockScore: 117.196
DOCK SCORE: 135.582
Gold.Goldscore.Fitness: 80.664
internal serial number: 11C3

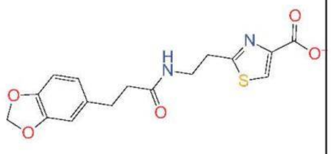

ID: 32942563
LibDockScore: 104.569
DOCK SCORE: 129.683
Gold.Goldscore.Fitness: 78.077
internal serial number: 11A2

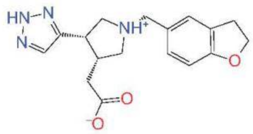

ID: 34006080
LibDockScore: 112.814
DOCK SCORE: 107.277
Gold.Goldscore.Fitness: 78.184
internal serial number: 12A2

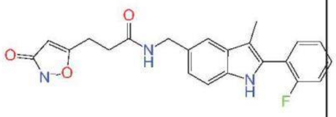

ID: 36498120
LibDockScore: 135.109
DOCK SCORE: 160.858
Gold.Goldscore.Fitness: 90.609
internal serial number: 11C5

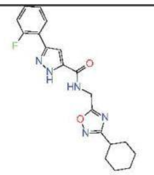

ID: 36752767
LibDockScore: 129.661
DOCK SCORE: 85.375
Gold.Goldscore.Fitness: 79.099
internal serial number: 11C8

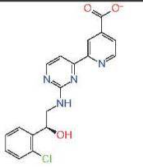

ID: 38603329
LibDockScore: 115.527
DOCK SCORE: 110.92
Gold.Goldscore.Fitness: 80.124
internal serial number: 12D2

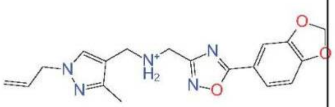

ID: 39206420
LibDockScore: 129.552
DOCK SCORE: 82.763
Gold.Goldscore.Fitness: 81.083
internal serial number: 11D4

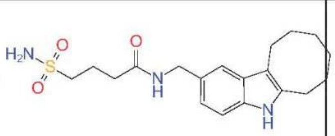

ID: 42422746
LibDockScore: 132.243
DOCK SCORE: 86.258
Gold.Goldscore.Fitness: 81.325
internal serial number: 13D7

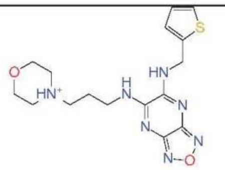

ID: 60419409
LibDockScore: 130.288
DOCK SCORE: 91.657
Gold.Goldscore.Fitness: 77.89
internal serial number: 11D2

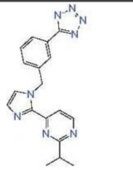

ID: 61117997
LibDockScore: 113.425
DOCK SCORE: 132.712
Gold.Goldscore.Fitness: 82.736
internal serial number: 12B6

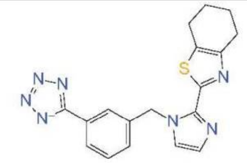

ID: 61802759
LibDockScore: 136.279
DOCK SCORE: 156.103
Gold.Goldscore.Fitness: 80.371
internal serial number: 12B1

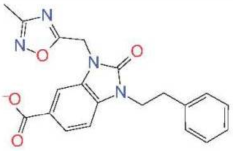

ID: 62571562
LibDockScore: 129.937
DOCK SCORE: 107.01
Gold.Goldscore.Fitness: 79.202
internal serial number: 11D5

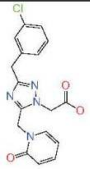

ID: 65156278
LibDockScore: 112.697
DOCK SCORE: 126.657
Gold.Goldscore.Fitness: 82.879
internal serial number: 12A7

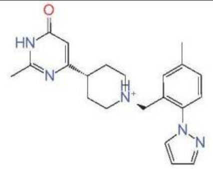

ID: 66646212
LibDockScore: 100.713
DOCK SCORE: 109.732
Gold.Goldscore.Fitness: 78.612
internal serial number: 13C2

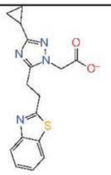

ID: 68589638
LibDockScore: 110.054
DOCK SCORE: 122.527
Gold.Goldscore.Fitness: 78.77
internal serial number: 12D7

Figure S2-C

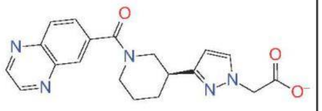

ID: 70715343
LibDockScore: 121.127
DOCK SCORE: 129.733
Gold.Goldscore.Fitness: 82.777
internal serial number: 12B5

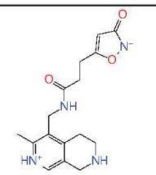

ID: 71401623
LibDockScore: 124.978
DOCK SCORE: 149.697
Gold.Goldscore.Fitness: 79.724
internal serial number: 11B7

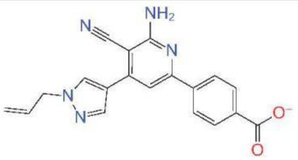

ID: 73835966
LibDockScore: 125.186
DOCK SCORE: 154.164
Gold.Goldscore.Fitness: 77.709
internal serial number: 12C5

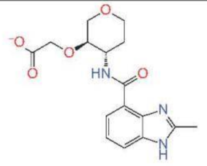

ID: 74345845
LibDockScore: 127.146
DOCK SCORE: 127.3
Gold.Goldscore.Fitness: 77.754
internal serial number: 11D7

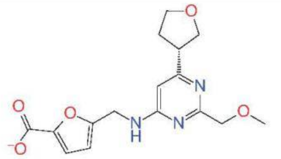

ID: 74681628
LibDockScore: 117.878
DOCK SCORE: 121.688
Gold.Goldscore.Fitness: 81.09
internal serial number: 12A3

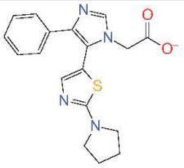

ID: 82085512
LibDockScore: 97.992
DOCK SCORE: 92.077
Gold.Goldscore.Fitness: 82.192
internal serial number: 13B1

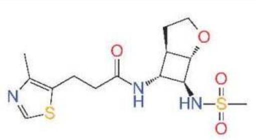

ID: 84833551
LibDockScore: 110.717
DOCK SCORE: 132.355
Gold.Goldscore.Fitness: 78.532
internal serial number: 12A6

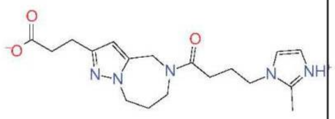

ID: 87347591
LibDockScore: 121.226
DOCK SCORE: 118.501
Gold.Goldscore.Fitness: 81.468
internal serial number: 13A6

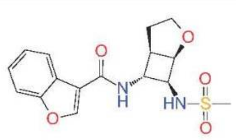

ID: 89326211
LibDockScore: 115.838
DOCK SCORE: 136.727
Gold.Goldscore.Fitness: 77.87
internal serial number: 12A5

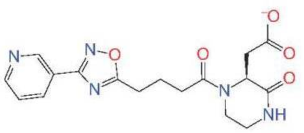

ID: 90017512
LibDockScore: 120.82
DOCK SCORE: 131.705
Gold.Goldscore.Fitness: 82.767
internal serial number: 13D3

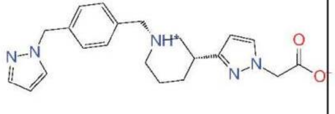

ID: 90531246
LibDockScore: 126.735
DOCK SCORE: 109.586
Gold.Goldscore.Fitness: 78.228
internal serial number: 12D8

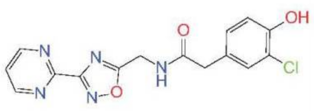

ID: 92371976
LibDockScore: 113.037
DOCK SCORE: 144.212
Gold.Goldscore.Fitness: 79.588
internal serial number: 13B2

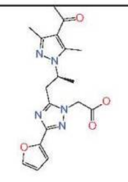

ID: 92647688
LibDockScore: 132.728
DOCK SCORE: 122.852
Gold.Goldscore.Fitness: 77.517
internal serial number: 11C1

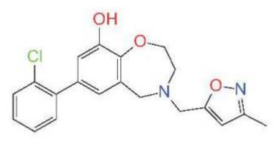

ID: 95609321
LibDockScore: 123.984
DOCK SCORE: 85.294
Gold.Goldscore.Fitness: 83.722
internal serial number: 11C6

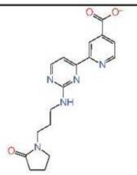

ID: 95966160
LibDockScore: 123.898
DOCK SCORE: 116.725
Gold.Goldscore.Fitness: 77.684
internal serial number: 13D6

Figure S2-D

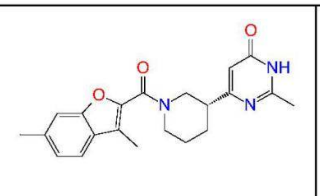

ID: 97067290
LibDockScore: 129.378
DOCK SCORE: 137.793
Gold.Goldscore.Fitness: 78.137
internal serial number: 13A8

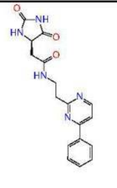

ID: 97369425
LibDockScore: 121.346
DOCK SCORE: 130.758
Gold.Goldscore.Fitness: 81.649
internal serial number: 11C2

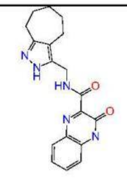

ID: 98064542
LibDockScore: 118.655
DOCK SCORE: 86.666
Gold.Goldscore.Fitness: 81.091
internal serial number: 12B7

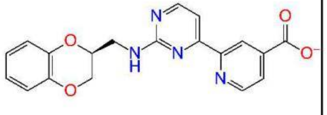

ID: 98863597
LibDockScore: 124.618
DOCK SCORE: 120.813
Gold.Goldscore.Fitness: 79.781
internal serial number: 12B8

Figure S2-E

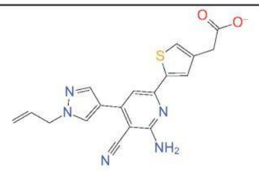

ID: 49319302
internal serial number: 21A1
Gold.Goldscore.Fitness: 83.965

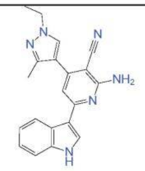

ID: 75316987
internal serial number: 21A2
Gold.Goldscore.Fitness: 72.694

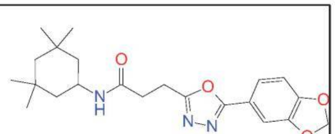

ID: 50207330
internal serial number: 21A3
Gold.Goldscore.Fitness: 59.005

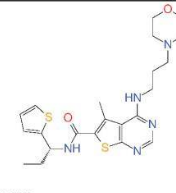

ID: 15951367
internal serial number: 21A4
Gold.Goldscore.Fitness: 79.581

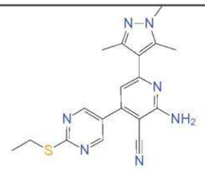

ID: 96758113
internal serial number: 21A5
Gold.Goldscore.Fitness: 66.15

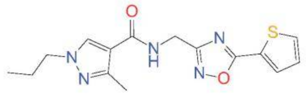

ID: 18652519
internal serial number: 21A6
Gold.Goldscore.Fitness: 63.591

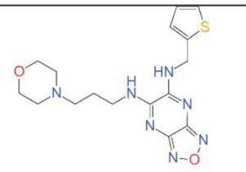

ID: 60419409
internal serial number: 21A8
Gold.Goldscore.Fitness: 80.416

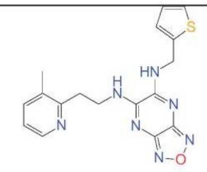

ID: 86039678
internal serial number: 21B1
Gold.Goldscore.Fitness: 75.452

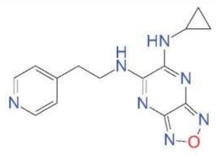

ID: 68809714
internal serial number: 21B2
Gold.Goldscore.Fitness: 74.147

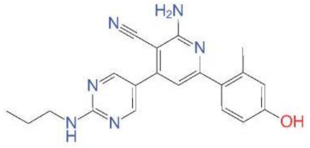

ID: 16057510
internal serial number: 21B3
Gold.Goldscore.Fitness: 80.015

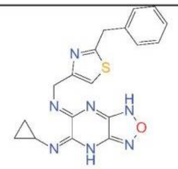

ID: 74424358
internal serial number: 21A7
Gold.Goldscore.Fitness: 75.576

Figure S3. The hydrophobic amino acid residues interacting with the ligand found by Interaction fingerprints analysis and the hydrophobic interaction diagram.

Figure S3-A The hydrophobic amino acid residues interacting with the compound 11D4

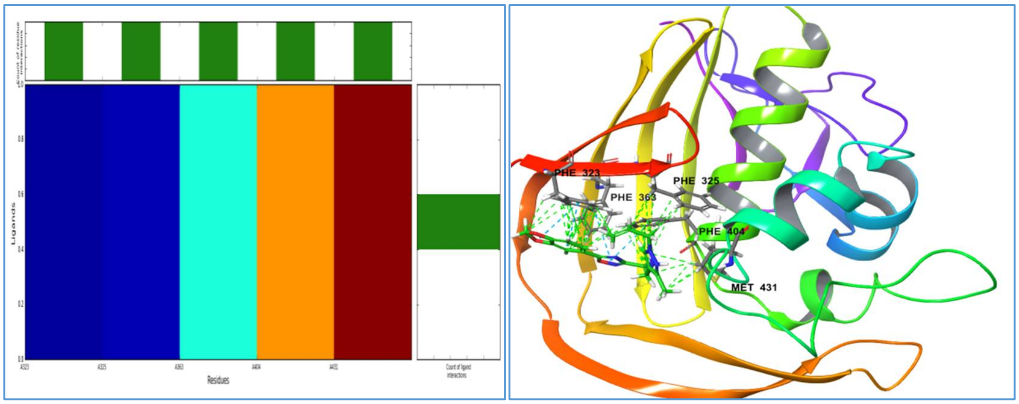

Figure S3-B The hydrophobic amino acid residues interacting with the compound 12C5

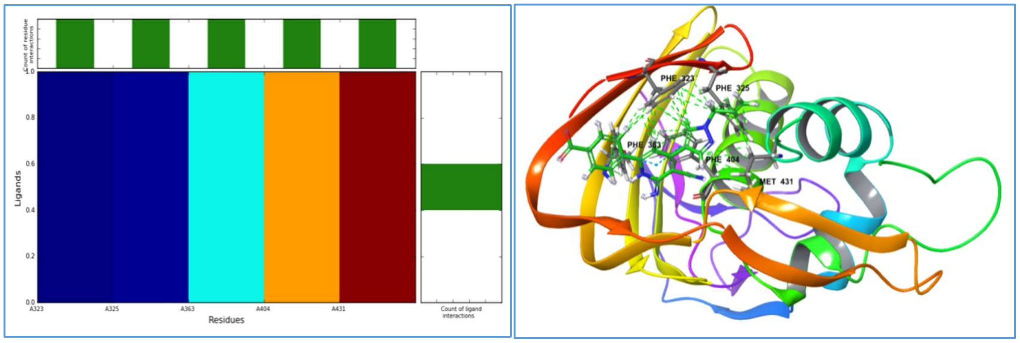

Figure S3-C The hydrophobic amino acid residues interacting with the compound 21A5

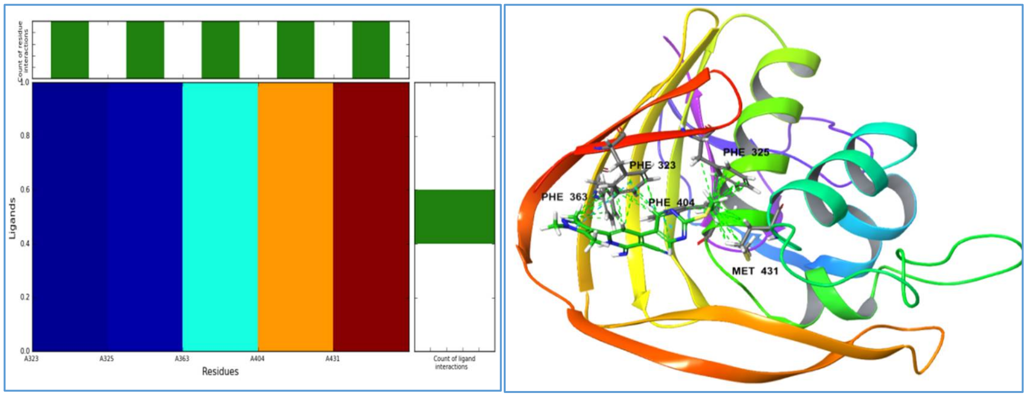

Figure S3-D The hydrophobic amino acid residues interacting with the compound 21B1

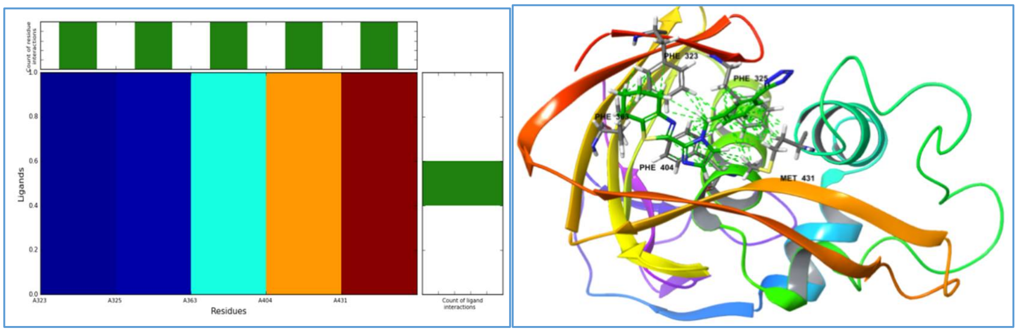

Figure S3-E The hydrophobic amino acid residues interacting with the VX-787

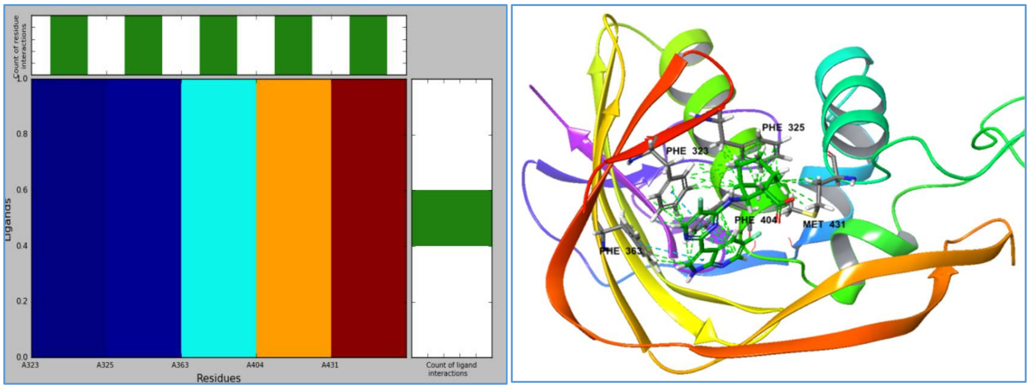

Figure S4. PB2 Cap-Binding Domain Sequence alignment of Influenza A/Victor/3/1975 (H3N2) and Influenza B Lee

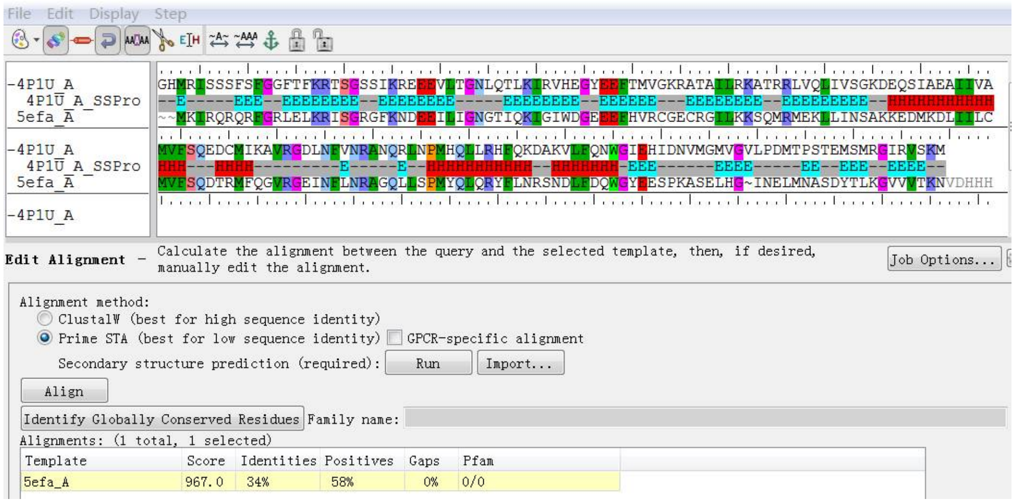

Figure S5. The sequence alignment results of the CBD site of influenza A virus and influenza B (partial) and the key amino acids that interact with active compounds.

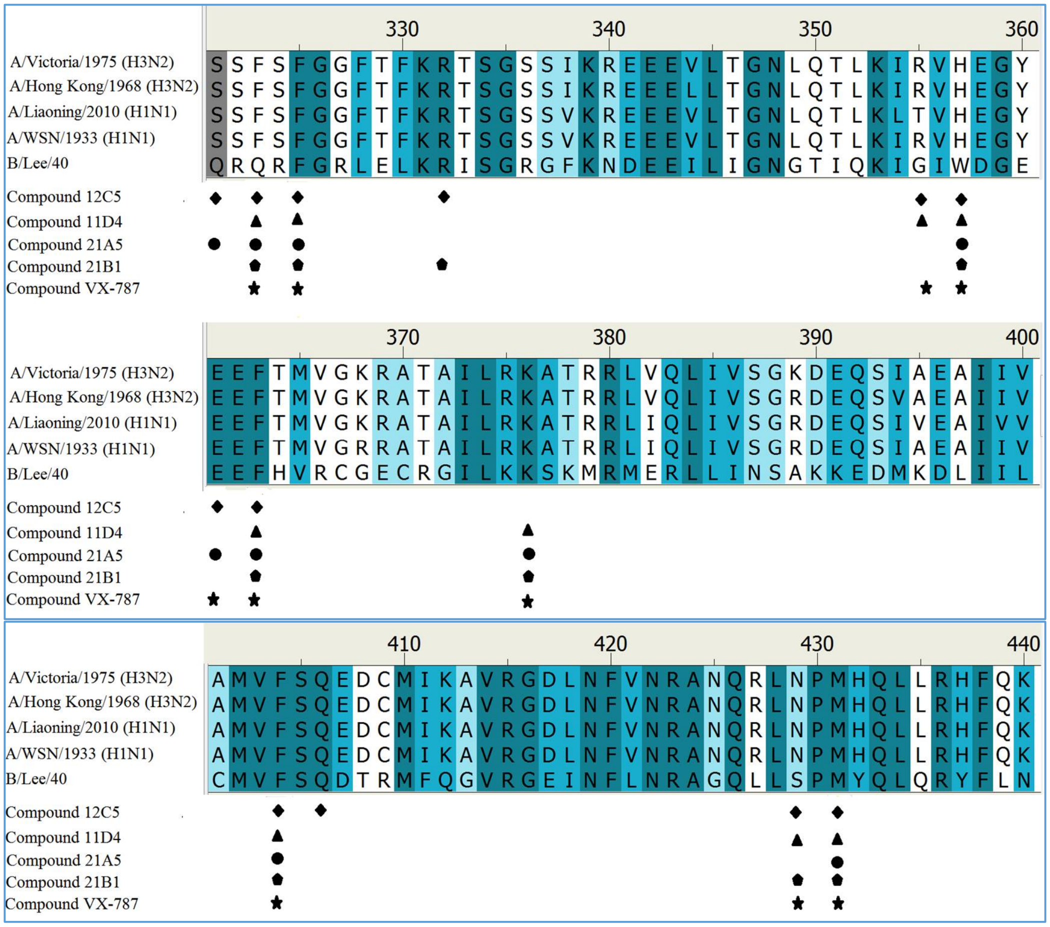

Table S2 The sequences of the primers

Primers	Sequence
190822-R1	GTGCTCGAGCTATCATTTAATCCACGCCCATCTTGCTTATAC
190822-F2	CTGGAAGTCCTGTTTCAGGGACCCCGTATTAGCAGCTCCTTTAGCT
190822-F3	GGGAACAGATTGGAGGACTGGAAGTCCTGTTTCAGGGAC

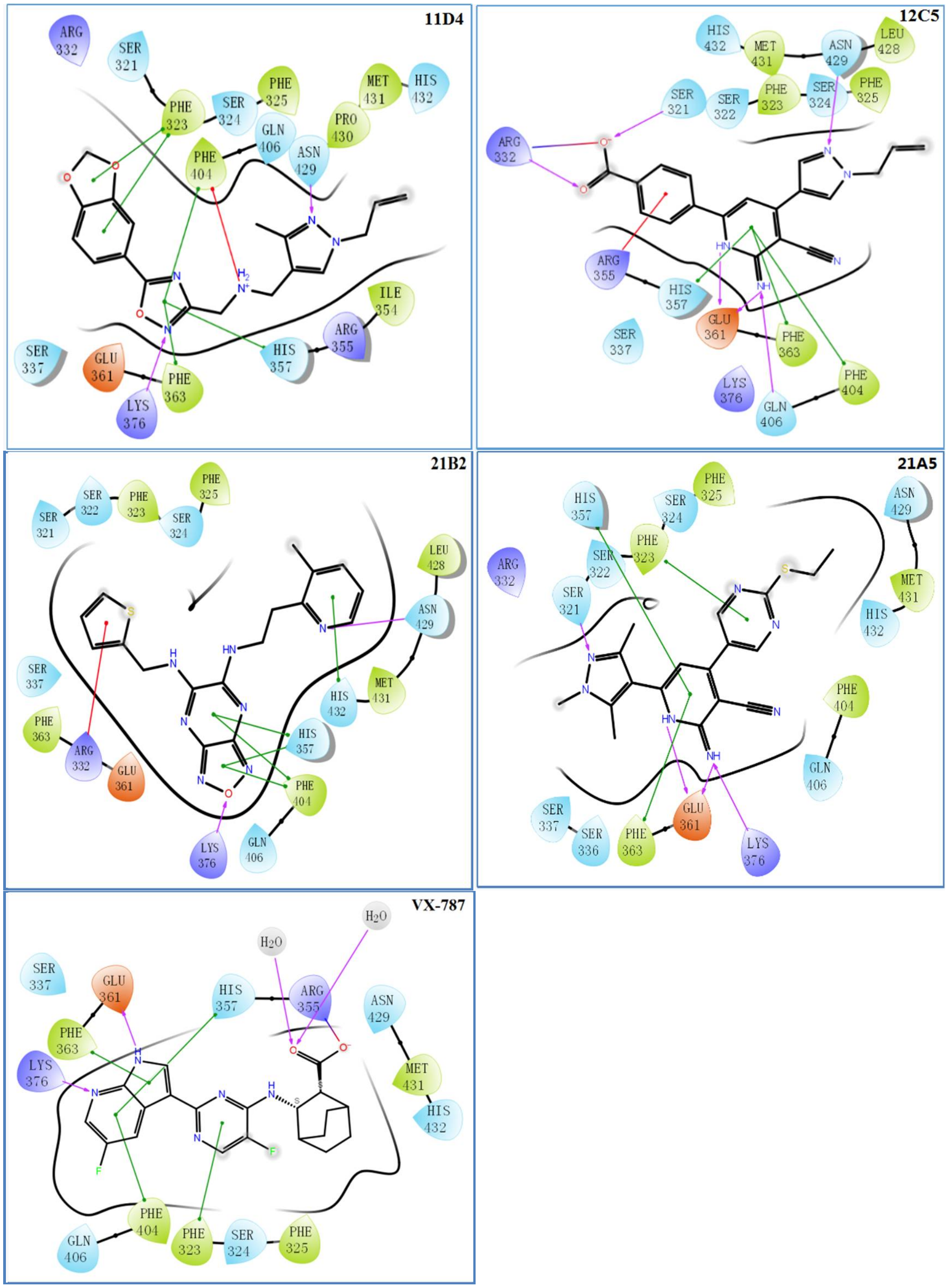

Figure S6. Ligand interaction diagrams of compounds 21B1, 11D4, 12C5, and 21A5.

Hydrogen bonds are represented by magenta arrows, π-π stacking is represented by forest-green lines, and salt bridges and cation-π stacking are represented by red lines.
